# SIFamide Influences Feeding in the Chagas Disease Vector, *Rhodnius prolixus*

**DOI:** 10.3389/fnins.2020.00134

**Published:** 2020-02-21

**Authors:** Mahnoor Ayub, Mariam Hermiz, Angela B. Lange, Ian Orchard

**Affiliations:** Department of Biology, University of Toronto Mississauga, Mississauga, ON, Canada

**Keywords:** insect, blood meal, RNAi, immunohistochemistry, neurohormone, heartbeat

## Abstract

SIFamides are a family of highly conserved neuropeptides in arthropods, and in insects are mainly expressed in four medial neurons in the pars intercerebralis of the brain. Although SIFamide has been shown to influence sexual behavior, feeding, and sleep regulation in holometabolous insects such as *Drosophila melanogaster*, little is known about its role in hemimetabolous insects, including the blood-sucking bug, *Rhodnius prolixus.* In this study, we confirm the nucleotide sequence for *R. prolixus* SIFamide (Rhopr-SIFa) and find characteristic phenotypic expression of SIFamide in four cells of the pars intercerebralis in the brain. In addition to extensive SIFa projections throughout the entire central nervous system, SIFamidergic processes also enter into the corpus cardiacum, and project along the dorsal vessel, suggestive of Rhopr-SIFa acting as a neurohormone. Physiologically, Rhopr-SIFamide induces dose-dependent increases in heartbeat frequency *in vitro* suggesting the presence of peripheral receptors, and thereby indicating Rhopr-SIFa is released to act upon peripheral targets. We also explore the function of Rhopr-SIFa in *R. prolixus*, specifically in relation to feeding, since *R. prolixus* is a blood-gorging insect and a vector for Chagas disease. The intensity of SIFamide-like staining in the neurons in the brain is diminished 2 h following feeding, and restocking of those cells is finished 24 h later, indicating Rhopr-SIFa may be released at feeding. The results of temporal qPCR analysis were consistent with the immunohistochemical findings, showing an increase in Rhopr-SIFa transcript expression in the brain 2 h after feeding. We also observed enhanced feeding (size of meal) in insects injected with Rhopr-SIFa whereas insects with RNAi-mediated knockdown of the Rhopr-SIFa transcript consumed a significantly smaller blood meal relative to controls. These data suggest that the four SIFamidergic neurons and associated arborizations may play an important function in the neuronal circuitry controlling *R. prolixus* feeding, with Rhopr-SIFa acting as a central and peripheral neuromodulator/neurohormone.

## Introduction

Neuropeptides constitute a functionally diverse class of neuroactive chemicals responsible for regulating physiological processes and behaviors such as ecdysis, reproduction, and feeding (Nässel, 2002; Nässel and Zandawala, 2019). An interesting family of neuropeptides that contribute to flexibility and diversity in physiological and behavioral regulation is the SIFamide family. First identified in the gray fleshfly *Neobellieria bullata*, SIFamides have since been observed across arthropods, including the malaria mosquito *Anopheles gambiae*, the beetle *Agelastica alni*, the moth *Galleria mellonella*, the honeybee *Apis mellifera*, the giant tiger prawn *Penaeus monodon*, the Jonah crab *Cancer borealis* and the red swamp crayfish *Procambarus clarkii* (Janssen et al., 1996; Sithigorngul et al., 2002; Huybrechts et al., 2003; Verleyen et al., 2004; Yasuda et al., 2004). In insects, SIFamides are conserved both in their distribution (present in two pairs of large diameters neurons in the brain) and in their sequence (Verleyen et al., 2009). Initially suggested to be restricted to holometabolous insects, i.e., insects with complete metamorphosis, SIFamides have now been found in insects that undergo incomplete metamorphosis, i.e., hemimetabolous insects. The presence of the peptide family has now been reported in the nervous system of the desert locust *Schistocerca gregaria* (Gellerer et al., 2015) and in the kissing bug, *Rhodnius prolixus* (Ons et al., 2009).

The role of SIFa as a neurohormone has been debated with initial reports maintaining it was only found within the central nervous system (CNS) of *Drosophila melanogaster* (Terhzaz et al., 2007; Gellerer et al., 2015). Lismont et al. (2018), however, found the SIFamide receptor transcript to be expressed in the periphery of *Bombus terrestris* suggesting that SIFamide may circulate in the hemolymph and control peripheral tissues. Although the physiological effects of SIFamide on peripheral tissues has not been explored, multiple functions for SIFamide have been reported in a variety of insects. In *D. melanogaster*, SIFamide has been shown to affect sexual behavior (Sellami and Veenstra, 2015), sleep regulation (Park et al., 2014) and pupal mortality (Agrawal et al., 2013). In the blacklegged tick *Ixodes scapularis*, Šimo et al. (2009, 2013) observed interactions between the SIFamide-signaling pathway and myoinhibiting peptide (MIP) pathway, and their involvement in feeding. SIFamide has been further implicated in modulating feeding behavior in *D. melanogaster* (Martelli et al., 2017) by enhancing odor guided appetitive behavior. The four characteristic SIFamidergic neurons in the pars intercerebralis of the brain have been suggested as integration centers for multiple neuronal inputs relaying signals for both satiety and hunger (Martelli et al., 2017). Little is known, about SIFamide’s role in hemimetabolous insects.

*Rhodnius prolixus*, a pioneer model organism in the study of insect physiology, is a blood-feeding hemipteran native to Central and South America. Significant amounts of blood are required for energy consuming activities that have an epidemiological relevance, such as reproduction, seeking food sources, and dispersion (Friend et al., 1965). Triatomines, such as *R. prolixus*, are hemimetablous insects and all post-embryonic stages consume a blood meal that in fifth instars may be equivalent to up to 10 times their unfed body weight. The five instar stages require this blood meal to trigger growth and development into the next instar or adult (Martini et al., 2007). Adults acquire energy from the blood meal to fuel other energy consuming behaviors such as mating and egg laying. Immediately after ingestion of a blood meal, *R. prolixus* triggers diuresis (excretion of urine) and associated downstream physiological events to remove excess water and salt (Orchard, 2006, 2009; Martini et al., 2007). During diuresis, *R. prolixus* transmits the protozoan parasite *Trypanosoma cruzi*, the disease-causing agent of Chagas disease, to a human host. Studying *R. prolixus* as a model for understanding triatomine feeding behavior and associated physiology provides fundamental knowledge for use in controlling these disease vectors.

In this study, we provide evidence for SIFamide’s role in regulating feeding-related physiology in *R. prolixus*. We also present immunohistochemical and physiological data suggesting SIFamide as a neurohormone.

## Materials and Methods

### Animals

Male and female fifth instar (6 weeks post-fed as fourth instars) and adult *R. prolixus* were taken from a colony maintained at 25°C and 60% humidity at the University of Toronto Mississauga. The insects were fed on defibrinated rabbit’s blood (Cedarlane Laboratories, Burlington, ON, United States) once in each instar.

### Verification of cDNA Sequence Encoding Rhopr-SIFamide

The complementary DNA (cDNA) for the coding sequence of *R. prolixus* SIFamide (as published by Ons et al., 2011) was uploaded into Geneious 8.1 (Auckland, New Zealand) from GenBank (Accession No.: ACT35307.1), and targets for 5′ and 3′ amplification by PCR were selected. In order to obtain the nucleotide sequence for the complete open reading frame (ORF) of Rhopr-SIFa, total RNA was extracted from the CNS of 5^th^ instar *R. prolixus* and cDNA was reversibly transcribed using the Applied Biosystems High Capacity cDNA Reverse Transcription Kit (Thermo Fisher Scientific Baltics UAB, Vilnius, Lithuania). The cDNA was used as template for PCR with Gene Specific Primers (GSPs) (Supplementary Table S1). OneTaq^®^ DNA Polymerase (NEB, Whitby, ON, Canada) was used for all PCRs. The cycling profiles for the PCRs using Bio-Rad’s s100 thermocycler (Bio-Rad Laboratories, Mississauga, ON, Canada) were: initial denaturation at 94°C for 5 min, followed by 29 cycles at 94°C for 30 s, 55–60°C annealing for 1 min, 68°C for 1 min and a final extension at 68°C for 5 min. Products from the reactions were gel extracted using EZ-10 Spin Column DNA Gel Extraction Kit (Bio Basic, Markham, ON, Canada) and cloned using pGEM-T Easy Vector (Promega, Madison, WI, United States). White colonies containing the inserts, tested using PCR, were inoculated and left overnight to grow. The inserts were extracted using EZ-10 Spin Column Plasmid DNA MiniPreps Kit (Bio Basic, Markham, ON, Canada) and sent for Sanger sequencing to Eurofins Genomics (Toronto, ON, Canada).

Gene specific primers (see Supplementary Table S1) were used (Sigma Aldrich Canada, Co., Oakville, ON, Canada) along with pDNR-LIB-Fw1 plasmid primer to amplify the cDNA regions at the 3′ end of the receptors (3′ Modified RACE). The product of the first reaction was nested and this process was repeated until one characteristic band for the transcript was observed. The band was gel purified, cloned and sequenced. Similarly, reverse GSPs (see Supplementary Table S1) were designed for the amplification of the 5′ cDNA end (5′ Modified RACE) with pDCR-LIB-Rev25. Essentially, a series of nested PCRs were utilized to distinguish the correct band. The products of the first reaction were purified and used as a template for the subsequent reaction. Selected fragments were gel extracted, cloned, and sequenced. The cDNA sequence for Rhopr-SIFa was analyzed against the sequence deposited in GenBank (Accession No.: ACT35307.1).

### Whole Mount Immunohistochemistry

Insects (fifth instars and adults) were dissected in physiological saline (150 mM NaCl, 8.6 mM KCl, 2 mM CaCl_2_, 4 mM NaHCO_3_, 34 mM glucose, 8.5 mM MgCl_2_, 5 mM HEPES, pH 7). Fixation and staining were performed as described by Mollayeva et al. (2018), but using a primary anti-SIFamide antiserum raised in rabbit (antiserum kindly provided by Dr. J. A. Veenstra, Bordeaux University, France) at a concentration of 1:10,000 in 0.4% Triton-X and normal goat serum (NGS) for 48 h at 4°C. The secondary antibody, a 1:600 Cy3-labeled sheep anti-rabbit immunoglobulin (Jackson ImmunoResearch Laboratories, West Grove, PA, United States) was made up in phosphate-buffered saline (PBS) containing 10% NGS for 24 h. Following washes in PBS, an ethanol series (starting with 25% ethanol diluted in distilled water, and increasing in 25% increments) was performed and tissues were mounted in methyl salicylate. Control tissues were prepared following the same protocol as above with the primary antiserum pre-absorbed overnight with 10^–5^ M Rhopr-SIFa prior to use (TYKKPPFNGSIFa; purchased from Genscript Laboratories Piscataway, United States).

Images were acquired using a confocal microscope LSM-800 (Carl Zeiss, Jena, Germany) and processed with the software Zeiss LSM Image Browser. Confocal images were analyzed using ImageJ Software (Ferreira and Rasband, 2012) to quantify changes in Rhopr-SIFa-like immunoreactivity from pre- to post-fed (2 and 24 h) insects. Staining intensity was determined by tracing the stained region of the medial neurons in the brain, and processes in the optic lobes (OLs) (lamina and medulla) and assigning grayscale values ranging from 0 (minimum intensity) to 255 (maximum intensity). To ensure accuracy, all images were treated consistently with respect to scale and number of slices in the Z-stacks. This experiment was repeated a second time with similar results.

### Quantitative Real Time PCR (qPCR) Analyses

Central nervous systems from 4 to 5 week unfed fifth instar *R. prolixus*, and also 2 h post-feed and 24 h post-feed insects, were dissected in RNase free phosphate-buffered saline and stored in RNA later solution (Ambion, Austin, TX, United States). The tissues were lysed and RNA extracted using the Total RNA Minipreps Superkit (Bio Basic Canada, Inc., Markham, ON, Canada). Synthesis of cDNA was done using the Applied Biosystems High Capacity cDNA Reverse Transcription Kit (Thermo Fisher Scientific Baltics UAB, Vilnius, Lithuania). The cDNA was used at a concentration of 5 ng together with ADVANCED qPCR Mastermix with SUPERGREEN (Wisent Biocentre, Saint-Jean-Baptiste, QC, Canada), to amplify Rhopr-SIFa transcript and reference gene transcripts (beta-actin and ribosomal protein 49). The primers used are listed in Supplementary Table S1. All primer efficiencies were > 90%. The reactions were completed on the CFX384 Real-Time system (Bio-Rad Laboratories, Ltd., Mississauga, ON, Canada). Temporal expression of Rhopr-SIFa transcript was determined using the delta-delta Ct method, calculating expression at each time point post-fed relative to pre-fed levels. Three biological replicates were performed, with three technical replicates for each condition in every trial including a negative control without template cDNA.

### Double-Stranded RNA (dsRNA) Synthesis

Two overlapping fragments of the DNA template for dsRNA synthesis were prepared with PCR by conjugating the T7 RNA polymerase promoter to the 5′ end (5′-taatacgactcactatagggaga-3′) on Rhopr-SIFamide or ampicillin resistant gene (ARG) as previously described (Lee et al., 2013) (see Supplementary Table S2). The PCR products were used as template for double stranded RNA (dsRNA) synthesis using the T7 Ribomax Express RNAi System (Promega, Madison, WI, United States). After synthesis, the dsRNA was precipitated with isopropanol and eluted in RNAase/DNAase free water. It was then quantified at 260 nm wavelength using nanodrop. The quality of the dsRNA products was verified by 1% agarose gel electrophoresis and kept at −80°C until use. Before injection, the dsRNA was diluted with RNAase free water at a concentration of 2 μg/μl.

### dsRNA Delivery

Fifth instar *R. prolixus* were immobilized on surgical wax and 2 μg of dsRNA for Rhopr-SIFa was injected into the thoracic/abdominal hemocoel at the base of the metathoracic legs using a 10 μL Hamilton syringe. Two groups, each consisting of 20 bugs, were used in this experiment as follows: dsSIFa-injected and the control double stranded ampicillin resistance gene (dsARG)-injected. All bugs were left for 1 h at room temperature to recover and then placed into an incubator at 28°C on a 12:12 h light/dark cycle. Mortality and behavior were monitored for 7 days following injection. Knockdown of the Rhopr-SIFa transcript was calculated on different days following injection using qPCR (Supplementary Figure S3).

### Feeding Assays

Fifth instar insects were injected with 1 μL 10^–4^ M Rhopr-SIFa or 1 uL physiological saline and weighed to record pre-fed weight. After injection, insects were kept in a chamber with 12:12 h light/dark cycle, and were fed 3 h after injection on defibrinated rabbit blood for 20 min. This is the typical time for blood-gorging (Mollayeva et al., 2018) and whilst diuresis is underway during this time, the insects do not eliminate any urine until feeding has ceased (Maddrell, 1964). Immediately after feeding, insects were weighed to measure the weight of the meal. They were weighed again at hourly intervals for 4 h to record the rate of diuresis. For both saline-injected and Rhopr-SIFa-injected groups, five insects that had a post-feed weight less than 0.1 g were removed from the final data set, since they were assessed as not having fed.

Insects were injected with 1 μL of 2 μg/μL dsSIFa or 1 μL of 2 μg/μL dsARG were weighed to record pre-fed weight and were kept in a 12:12 h light/dark cycle chamber. Insects were fed 2 days following injection (the day with 92% Rhopr-SIFa transcript knockdown) on defibrinated rabbit blood for 20 min. As previously described, immediately after feeding insects were weighed to measure the size of the meal. They were weighed again at hourly intervals for 4 h to record the rate of diuresis.

### Heart Contraction Assay

The ventral abdominal cuticle and visceral tissues were removed from fifth instar *R. prolixus*, leaving the dorsal vessel exposed and the semi-intact preparations of the abdominal region of the dorsal vessel and surrounding dorsal cuticle were secured in a dish using minuten pins. Heart rate was measured in saline and in various doses of Rhopr-SIFa using electrodes connected to an impedance converter (UFI model 2991, Morro Bay, CA, United States) and visualized on a computer using Picoscope 2200 (Pico Technology, St Neots, United Kingdom). Electrodes were placed on either side of the heart between the sixth and seventh abdominal segments. Preparations were maintained in 100 μL saline, and test solutions were added by replacing 50 μL of saline with 50 μL of peptide.

### Statistical Analyses

All graphs were created with GraphPad Prism 7^1^. Immunoreactivity was quantified using ImageJ and analyzed using a one-way ANOVA followed by the Tukey’s *post hoc* test to determine intensity differences over time after feeding. Heart contraction assay data was analyzed with a Student’s *t*-test to determine differences in contraction frequency relative to saline after application of a range of Rhopr-SIFa concentrations. Rhopr-SIFa transcript expression using qPCR was analyzed with a one-way ANOVA followed by a Tukey’s *post hoc* test to compare expression at each time point relative to unfed expression in the brain. Feeding assay data was also analyzed with Student’s *t*-test to compare post-fed body weights between treatment and control groups. Rate of weight loss after feeding, as an indicator of rate of diuresis, was analyzed by fitting the data with regression lines and comparing the slopes for significant differences using an *F*-test.

## Results

### Verification of Rhopr-SIFa Prepropeptide and Nucleotide Sequence

**FIGURE 1:**
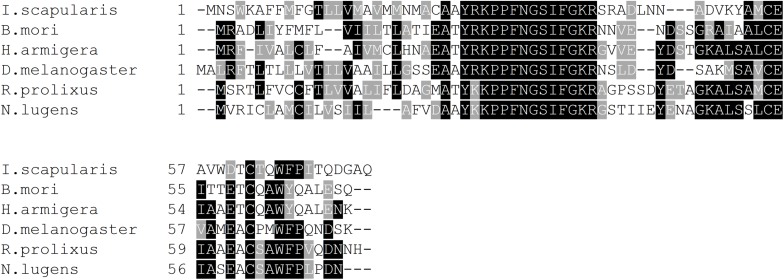
Multiple sequence alignment of arthropod SIFamides generated by Clustal Omega tool. Identical and similar amino acids across 60% of the sequences are shaded in black and gray, respectively. Short form and GenBank accession numbers are indicated for each species. *D. melanogaster, Drosophila melanogaster* (NP_001246496.1); *B. mori, Bombyx mori* (BAG50362.1); *N. lugens, Nilaparvata lugens* (BAO00977.1); *I. scapularis, Ixodes scapularis* (ADD92393.1); *H. armigera, Helicoverpa armigera* (AGH25569.1).

Isolating the SIFa transcript from *R. prolixus* CNS cDNA (Supplementary Figure S1) confirms the Rhopr-SIFa sequence reported by Ons et al. (2011). We have sequenced 608 base pairs of Rhopr-SIFa cDNA, where the ORF starts at base 171, and ends at base 393, yielding a prepropeptide consisting of 74 amino acids. The nucleotide sequence of Rhopr-SIFa was used in a BLASTN search against the *R. prolixus* preliminary genome assembly database using Geneious Pro 8.0.4 software (Kearse et al., 2012), which predicted 3 exons and 2 introns in Rhopr-SIFa (Supplementary Figure S1), spanning approximately 2.8 kb of the whole genome. The signal peptide, as predicted by SignalP and similar to that reported by Ons et al. (2011), is 24 amino acids long, with a cleavage site predicted between residues AMA-TY. The Rhopr-SIFa precursor amino acid sequences from *R. prolixus* (Ons et al., 2011; GenBank Accession No. ACT35307.1) and other arthropods including *D. melanogaster* (NP_001246496.1), *B. mori* (BAG50362.1), *N. lugens* (BAO00977.1), *I. scapularis* (ADD92393.1), *H. armigera* (AGH25569.1) were aligned using Clustal W (Larkin et al., 2007) (Figure 1). In these arthropods, the nucleotide sequence encoding the SIFamide amino acid sequence has been confirmed by *in vitro* sequencing. The aligned prepropeptide sequences of identical and similar consensus sequences are denoted with black and gray, respectively, by using the BOXSHADE3.21 server^2^ and illustrate the conserved nature of the SIFamide family (Figure 1). Confirming the mature Rhopr-SIFa sequence allows us to use qPCR, RNA interference and Rhopr-SIFa for physiological assays and as a control for immunohistochemistry.

### SIFamide-Like Immunoreactivity in the CNS, Corpus Cardiacum, and Dorsal Vessel

**FIGURE 2:**
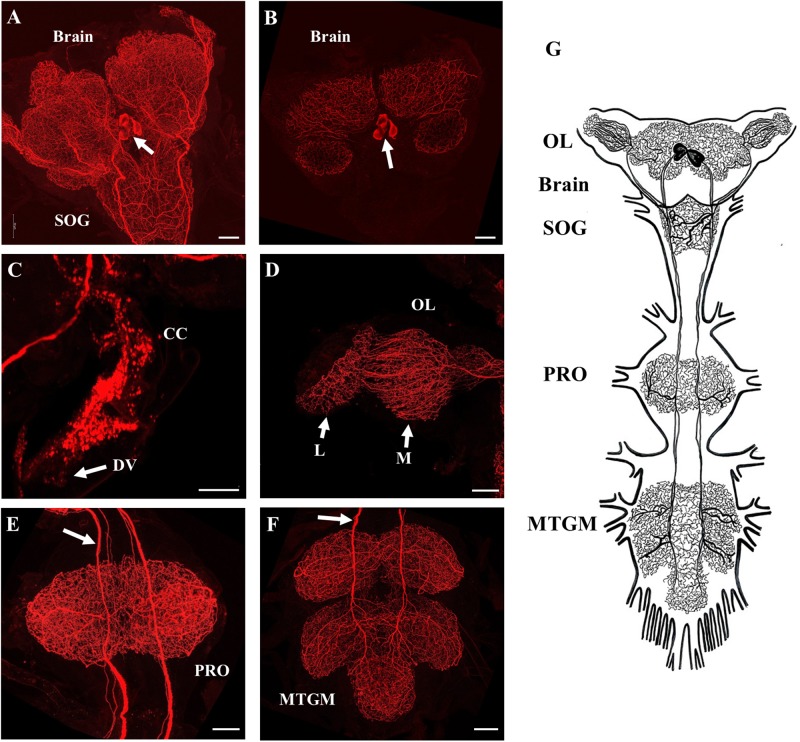
SIFamide-like immunoreactivity (SLI) in the central nervous system (CNS) of fifth instar *R. prolixus.* The staining pattern seen here was the same for male and female fifth instar *R. prolixus*. **(A)** Ventral view of the brain and the sub-esophageal ganglion (SOG), cell bodies indicated by arrow. **(B)** Dorsal view of the brain showing the two pairs of cell bodies (arrow). **(C)** SLI in processes in the corpus cardiacum (CC) extending onto the dorsal vessel (DV). **(D)** Projections from the SIFamidergic neurons extending into the optic lobe (OL), medulla (M), and lamina (L). **(E)** View of the prothoracic ganglion (PRO) with axons continuing from the SOG. The arrow points to separations in the axons that exist as closely oriented pairs. **(F)** View of the mesothoracic ganglionic mass (MTGM) with axons (arrow) continuing from the PRO. **(G)** Diagrammatic representation of the two pairs of cell bodies and their extreme projections throughout all ganglia. Map is drawn from over 20 preparations. Scale bars = 50 μm.

SIFamide-like immunoreactivity (SLI) was found in the CNS as previously reported by Ons et al. (2011) but here processes are shown to project into the corpus cardiacum (CC) and along the dorsal vessel of fifth instar *R. prolixus* (Figure 2). Two pairs of neurons in the pars intercerebralis of the brain stained brightly (Figures 2A,B) with axons projecting throughout the ventral nerve cord with extensive processes in the neuropiles of the brain and all ganglia of the ventral nerve cord (Figures 2A–F). Processes also extended from these cells into the CC and dorsal vessel (Figure 2C) as well as the medulla and lamina of the OL (Figure 2D). Each neuropile has a dense meshwork of SIFa-like immunoreactive processes. The composite diagram in Figure 2G illustrates the extensive distribution of processes originating from the four neurons in the brain.

The distribution of SLI was consistent in both male and female fifth instar *R. prolixus*. No SLI was observed on the salivary glands, foregut, anterior midgut, posterior midgut, or hindgut. Control tissues incubated in antiserum preabsorbed with Rhopr-SIFa had markedly reduced staining intensity of cell bodies in the brain with elimination of staining in the two pairs of axons and neuropils (Supplementary Figure S2).

### SIFamide-Like Immunoreactivity and Transcript Expression in the CNS After Feeding

**FIGURE 3:**
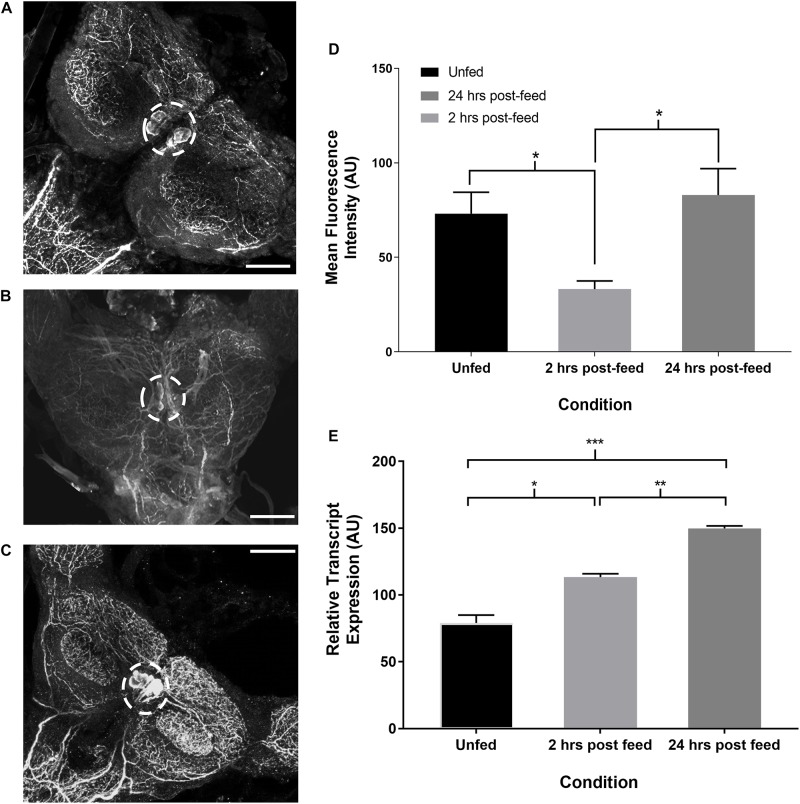
Time-course intensity of SLI in 5^th^ instar *R. prolixus* brain. Confocal images showing SLI in **(A)**: unfed, **(B)**: 2 h post-feed and **(C)**: 24 h post-feed conditions. **(D)** Mean fluorescence intensity (grayscale values, arbitrary units) in the area of cell bodies in the brain relative to time of feeding. There is a significant decrease in total fluorescence in the neurons from unfed (*n* = 9) to 2 h post-feed (*n* = 8) (Student’s *t*-test, *p ≤ 0.05). 24 h after feeding (*n* = 10) the cells have restocked (Student’s *t*-test, *p < 0.05). Error bars represent standard error of the mean. **(E)** Temporal expression of the Rhopr-SIFa transcript in the brain of 5^th^ instar *R. prolixus*. Expression significantly increases in the brain 2 h after feeding (one-way ANOVA, *p < 0.05) relative to expression in the unfed insect, and is again increased at 24 h post-feeding (one-way ANOVA, **p < 0.01, ***p < 0.001). Error bars represent standard error of the means from three trials. Scale bars = 50 μM.

Time-course immunohistochemistry on fifth instar CNS revealed diminished SIFamide-like staining in the two pairs of cell bodies in the brain (Figure 3) and varicosities extending over the protocerebrum in both lobes of the brain 2 h post-feeding (Figure 3B). Twenty-four hours post-feeding, SLI in the cell bodies suggests restocking, with intensity of staining in the cell bodies in the brain increasing their SLI relative to 2 h post-feeding levels (Figure 3C). Fluorescence intensity of cell bodies, quantified with grayscale values, was significantly diminished 2 h after feeding but had recovered their fluorescence 24 h after feeding (Figure 3D). Staining of neuronal processes in the sub-esophageal ganglion (SOG) appeared to diminish 2 h after feeding when compared to pre-feed levels (not shown). Twenty-four hours later, staining resembled pre-feed levels (Figure 3C). Expression of the Rhopr-SIFa transcript increased significantly in the brain at both 2 and 24 h after feeding, relative to expression in the brain of unfed insects (Figure 3E) indicative of restocking of the peptide.

**FIGURE 4:**
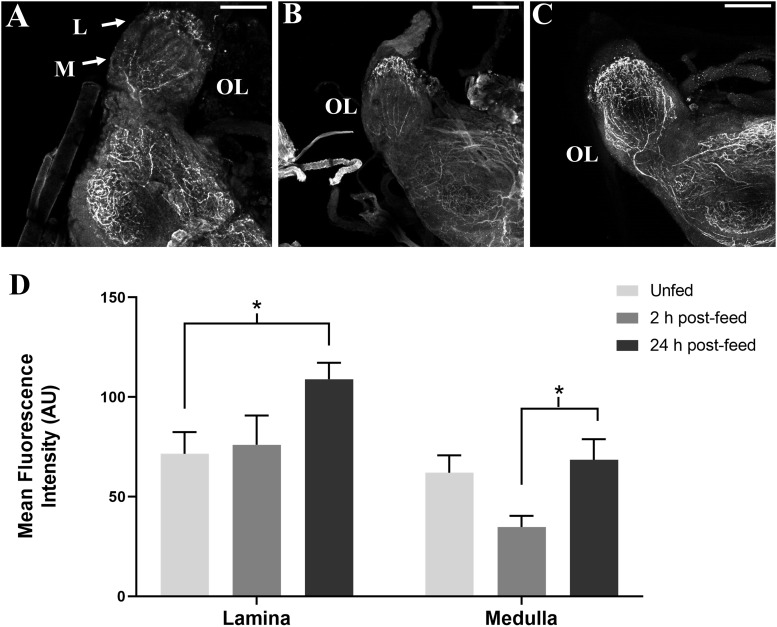
Time-course intensities of SLI in 5^th^ instar *R. prolixus* OL. Confocal images of the OL showing SLI in **(A)**: unfed, **(B)**: 2 h post-feed and **(C)**: 24 h post-feed conditions. **(D)** Mean fluorescence intensity (grayscale values, arbitrary units) in the lamina (L) and medulla (M) in the OL relative to time of feeding. There is a significant increase in total fluorescence in the lamina of the brain from unfed (*n* = 5) to 24 h post-feed (*n* = 6) (Student’s *t*-test, *p < 0.05) and a significant increase in fluorescence in the medulla from 2 h post-feed to 24 h post-feed conditions (*n* = 5), (Student’s *t*-test, *p < 0.05). Error bars represent standard error of the mean. Scale bars = 50 μM.

SIFamide-like immunoreactivity also decreased in the OL 2 h after feeding (Figure 4). Staining of neuronal processes in the lamina (L) appears to be unchanged 2 h after feeding, but increased in the 24 h post-feed condition relative to both unfed and 2 h post-feed states. Fluorescence intensity appears to decrease in the medulla (M) 2 h post-feeding (Figure 4D) returning to the level in the unfed condition 24 h after feeding (Figure 4C).

### Feeding Assays

**FIGURE 5:**
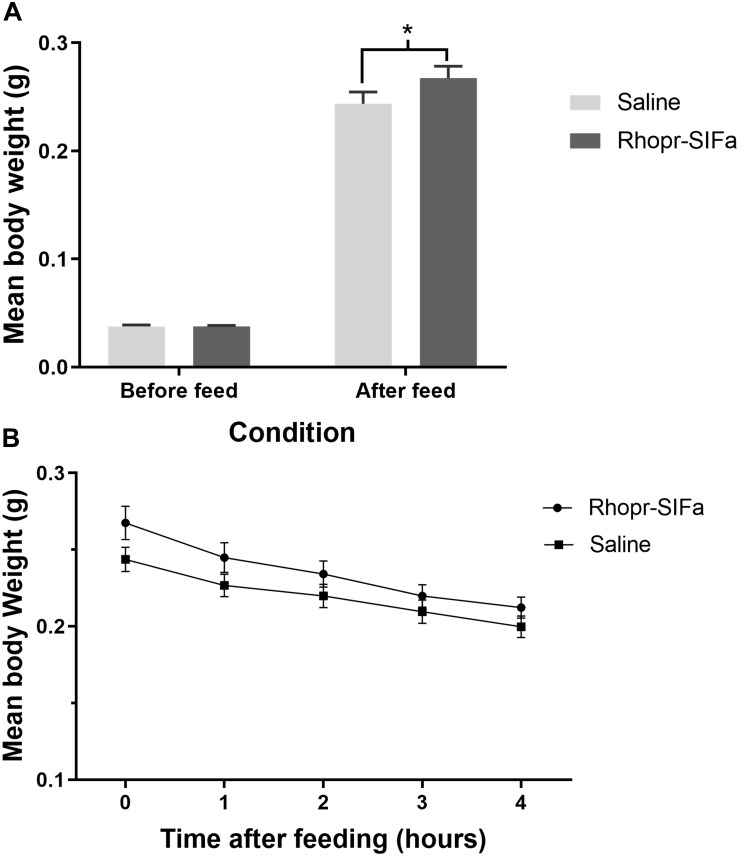
The effect of Rhopr-SIFa injection on feeding and rate of diuresis in *R. prolixus*. **(A)** Bar chart of insects fed 3 h after injection with 10^–4^ M Rhopr-SIFa showing a significantly higher post-feed weight relative to saline injected controls (Student’s *t*-test, *p < 0.05, *n* = 15). **(B)** For insects fed 3 h after injection, rate of diuresis is not significantly different between the two groups by fitting the data using linear regression and analyzing the slopes for significance using an *F*-test (*p* > 0.05). Data points show mean ± SE (*n* = 15). This experiment was repeated a second time with similar results.

Fifth instar *R. prolixus*, injected with 1 uL of 10^–4^ M Rhopr-SIFamide 3 h prior to feeding, consumed a larger blood meal, as evidenced by significantly higher post-feed weight relative to saline injected controls (Figure 5A). Rhopr-SIFa-injected insects also appeared to exhibit more aggressive feeding behavior in comparison with saline-injected control insects. Insects fed in groups of 20 for 20 min 3 h post-injection, at which point feeding was terminated when insects removed their proboscises from the membrane and started moving about. Rhopr-SIFa-injected insects continued probing behavior even after their guts were fully inflated. The rate of weight loss over 4 h, a measure of diuresis, was not significantly different (Figure 5B) between the Rhopr-SIFamide-injected insects versus saline-injected controls (*p* > 0.05).

### RNAi-Mediated Knockdown of Rhopr-SIFa

Rhopr-SIFamide transcript levels were reduced by 92% in the brain 2 days after dsRNA injection in unfed fifth instar insects (Supplementary Figure S3). Immunohistochemical analysis verified the reduction in peptide level caused by injection of dsSIFa. Compared to the dsARG control group, the staining of cell bodies and processes containing SLI was either absent or reduced in the group injected with dsSIFa (Supplementary Figure S4).

**FIGURE 6:**
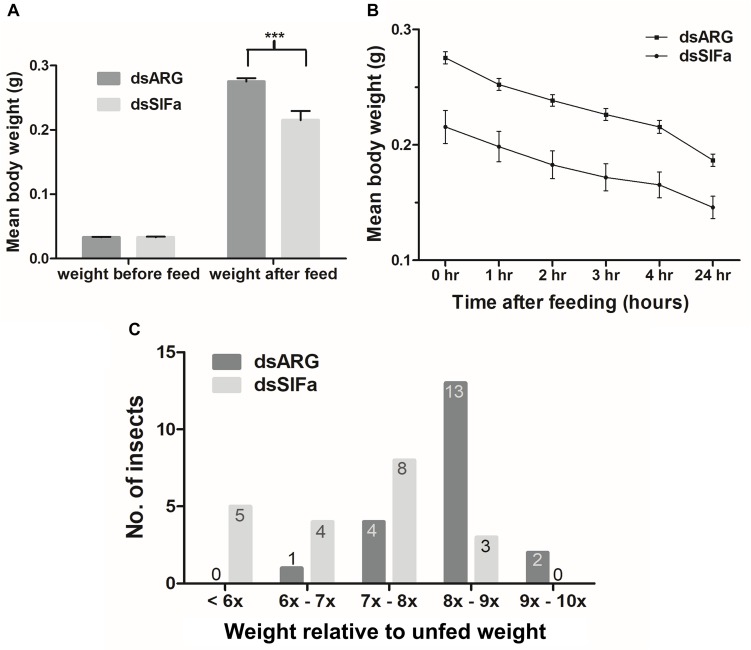
The effect of knockdown of Rhopr-SIFa transcript on *R. prolixus* feeding using double stranded RNA (dsRNA). Insects were injected with dsSIFa and were fed on day 2 when Rhopr-SIFa transcript expression level was 92% less relative to dsARG-injected controls. Error bars represent standard error of the mean. **(A)** Insects injected with dsSIFa feed significantly less than dsARG-injected controls. Asterisks indicate significant differences compared to the control group (unpaired *t*-test, ***p < 0.001) (*n* = 20 dsSIFa, *n* = 20 dsARG). **(B)** Weights of dsARG and dsSIFa-injected insects (injection 2 days prior to feeding) over the course of 4 and 24 h after feeding to depict diuresis. Rates of weight loss did not differ significantly between treatment and control groups, as determined by fitting the data using linear regression and analyzing the slopes for significance using an *F*-test (*p* > 0.05). **(C)** Number of insects for each interval of fed body weight relative to unfed weight, where “x” represents “n” multiple of unfed body weight. Note that 45% of insects injected with dsSIFa took less than 7X their unfed weight in blood versus 5% of those injected with dsARG.

Fifth instar *R. prolixus*, injected with 2 μg dsSIFa 2 days prior to feeding, consumed a smaller blood meal as seen by significantly lower post-feeding weight relative to dsARG-injected controls (Figure 6A). Although both groups (*n* = 20) were presented with a feeding stimulus for the same amount of time (i.e., 20 min), insects injected with dsSIFa appeared to remove their proboscises earlier than the dsARG-injected insects and began to move about in their jar. The rate of weight loss, a measure of diuresis, did not differ between dsARG and dsSIFa-injected insects (Figure 6B). After consuming a blood meal, 25% of insects injected with dsSIFa weighed less than 6X their unfed weight, compared to no insects in the dsARG control group (Figure 6C). For the dsARG-injected insects a majority (75% of insects) fed over 8–9X their unfed body weight, whereas for dsSIFa-injected insects a majority (85%) of the insects fed less than 8X their unfed body weight.

### The Effect of Rhopr-SIFamide on Heart and Dorsal Vessel Contractions

**FIGURE 7:**
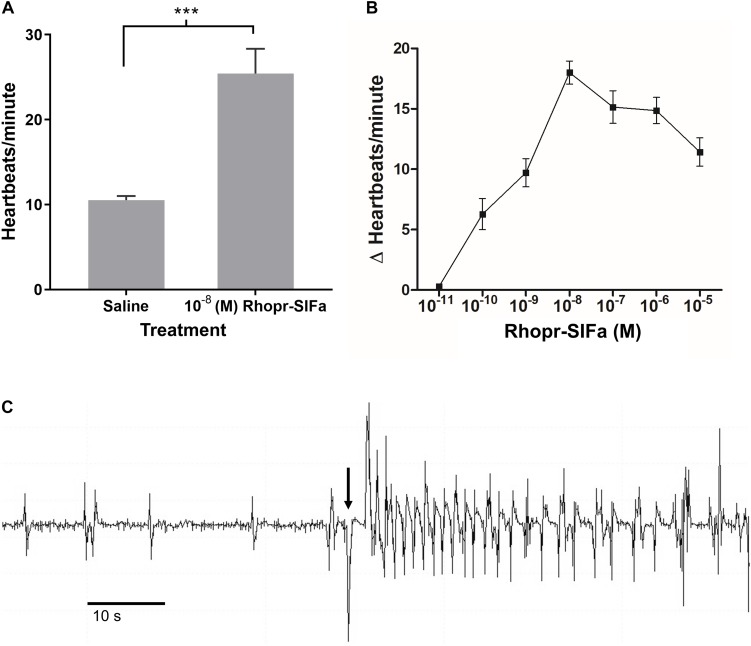
The effect of Rhopr-SIFa on heartbeat rate of fifth instar *R. prolixus*. **(A)** Student’s *t*-test reveals a significant difference between heartbeats/minute in 10^–8^ M Rhopr-SIFa relative to saline (***p < 0.0001), Mean + SE (*n* = 7). **(B)** Rhopr-SIFa stimulates a dose-dependent increase in the average frequency (Mean ± SE) of heart contractions of *R. prolixus*. **(C)** A sample trace, where the arrow indicates the application of 10^–8^ M Rhopr-SIFa that results in an increase in frequency of heart contractions (vertical deflections) relative to saline.

Since SLI is present in processes of the neurohemal organ, the CC, and along the dorsal vessel of *R. prolixus* we sought evidence that Rhopr-SIFa could have a physiological effect on a peripheral tissue. Spontaneous contractions of the heart begins with contraction of the alary muscles attached to the ventral surface of the dorsal vessel in the abdominal segments thereby expanding the heart chamber. Alary muscle contraction followed by relaxation initiates heart contractions, with contractions subsequently traveling anteriorly along the dorsal vessel. Rhopr-SIFa significantly increases heartbeat frequency in a reversible, dose-dependent manner (Figure 7). Threshold is between 10^–11^ M and 10^–10^ M with maximum at 10^–8^ M and an EC_50_ of approximately 8 × 10^–10^ M (Figure 7B). An example of an increase in heartbeat frequency due to application of 10^–8^ M Rhopr-SIFa is shown in Figure 7C.

## Discussion

The aim of this study was to investigate the possible role of Rhopr-SIFa, a member of a highly conserved family, in feeding in *R. prolixus*. We also show the presence of SLI in processes in the CC and along the dorsal vessel, suggesting peripheral actions of Rhopr-SIFa and possibly a neurohormonal function; providing a rationale for the existence of the SIFa receptor (SIFaR) in peripheral tissues as discussed in Lismont et al. (2018). The complete Rhopr-SIFa cDNA sequence from *R. prolixus* was cloned, matching, and extending the previously identified Rhopr-SIFa mRNA sequence (GQ253922.1) provided by Ons et al. (2011). The mRNA encodes a 74 amino acids long precursor peptide, including a signal sequence and the mature Rhopr-SIFa. The 12 amino acid long Rhopr-SIFa is highly conserved with a C-terminal amidation. The intensity of SLI and transcript expression was altered by feeding. We also observed altered feeding behavior in fifth instars injected with Rhopr-SIFa, exhibiting an agitated pattern of movement, and consuming a significantly larger blood meal. Likewise, insects injected with dsRNA for Rhopr-SIFa took in a significantly smaller blood meal. Interestingly, physiological assays involving application of Rhopr-SIFa on fifth instar dorsal vessel resulted in a dose-dependent increase in heartbeat. These results demonstrate a multifunctional role for Rhopr-SIFa in mediating feeding physiology and including a peripheral and possible neurohormonal role in *R. prolixus*.

SIFamide-like immunoreactivity has previously been shown to be localized in the CNS of *R. prolixus* (Ons et al., 2011), and our results verify the strong staining present in four SIFamidergic cells in the pars intercerbralis. A novel finding is the observation of SLI in neurohemal release sites in the CC and staining in processes along the dorsal vessel, suggesting additional release sites in the thorax. We also note the extensive network of SIFamide-like immunoreactive arborizations throughout the CNS, with two pairs of axons originating from neurons in the pars intercerebralis, and resulting in dense neuropils in each ganglia. There were no obvious differences in SLI between male and female fifth instar *R. prolixus*, suggesting a conserved role in integrating physiology common to both male and female insects.

The OLs also contained extensive SLI, similar to observations for *N. bullata* and *A. gambiae* (Verleyen et al., 2004). The presence of extensive SLI in the OLs of *R. prolixus* may suggest a potential function in mediating rhythmic behavior guided by environmental cues such as locomotion, feeding, and ecdysis (Davey, 2007). In *P. americana*, in addition to the conserved cells in the pars interecerebralis, SIFamidergic cells were also seen in the OLs and posterior protocerebrum (Arendt et al., 2016). Neuronal circuits for SIFamide seemed to be co-localized with pigment-dispersing factor, histamine, and gamma amino butyric acid, suggesting that SIFamide may be involved in regulating rhythmic behavior associated with the circadian system (Arendt et al., 2016). Similarly, immunohistochemical data from *D. melanogaster* also reveals synapses between the four SIFamidergic neurons and DN1 dorsal clock circadian pacemaker neurons (Cavanaugh et al., 2014), suggesting SIFamide may constitute an element in modulating behaviors governed by the circadian clock. In *R. prolixus*, since sensitivity to odors can be temporally modulated depending on the time of day and the type of signal, studying neuropeptides regulating these rhythmic responses can provide insight for endogenous signals governing the life history of the insect. The extensive presence of SIFamide in the CNS, coupled with abundant varicosities throughout the ganglia, may further indicate the peptide’s potential role in regulating adult functions such as reproduction, as suggested by Verleyen et al. (2004). Since the suppression of the SIFamide signaling pathway in *D. melanogaster* resulted in the modulation of sexual behavior (Terhzaz et al., 2007), SLI in the OLs may suggest a similar role for Rhopr-SIFa in *R. prolixus*. These results are in agreement with those seen in the *S. gregaria* CNS. Ramifications of varicose SIFamide-like immunoreactive processes indicate of axon terminal outputs (Gellerer et al., 2015), and their presence in the central complex suggests involvement in the selection and coordination of motor functions and spatial orientation (Pfeiffer and Homberg, 2014).

Here, we also show SLI in the cells and neuronal processes in the CNS is significantly diminished 2 h after feeding and increases 24 h later. These changes in fluorescence intensity suggest that the peptide is released and subsequently restocked in association with seeking and consuming a blood meal. The increase in Rhopr-SIFa transcript expression 2 and 24 h after feeding correlates with the change in fluorescence of SLI in the *R. prolixus* CNS, suggesting increased transcription of the Rhopr-SIFa gene to restock the cells. We also observed diminished staining in the medulla layer of the OL and increased SLI in the laminal neuropil, 2 h after feeding. Staining in both lamina and medullary neuropils increases 24 h after feeding. In *S. gregaria* and *D. melanogaster*, SLI has been observed in sensory neuropils of different modalities such as in OLs, antennal lobes, and the tritocerebral glomerular lobe (Verleyen et al., 2004; Terhzaz et al., 2007; Gellerer et al., 2015). Therefore, it can be suggested that Rhopr-SIFa may be mediating the interface between different sensory modalities and eliciting behaviors guided by visual, tactile or olfactory cues, triggering SIFamide release.

The link between Rhopr-SIFa release and feeding is confirmed in our feeding assays, whereby increasing the titer of Rhopr-SIFa in the hemolymph by injection induced a significantly larger blood meal to be consumed. In *D. melanogaster*, silencing SIFaR via RNAi has been linked to shorter sleep phenotypes (Park et al., 2014), and suggests a behavioral relevance for this signaling system. In *D. melanogaster*, starvation also appeared to increase activity of SIFamidergic neurons making the insect more sensitive to sensory stimuli (Martelli et al., 2017). In the present study, the transcript coding for Rhopr-SIFa was successfully knocked down via injection of its dsRNA in fifth instars. Knocking down the Rhopr-SIFa transcript in *R. prolixus* led to a significant decrease in the overall size of the blood meal consumed, where 85% of the insects injected with dsSIFa fed less than eight times their initial body weight, compared to only 25% in the dsARG-injected controls. Rhopr-SIFa would appear to promote blood-gorging in unfed *R. prolixus.*

The results of the dorsal vessel contraction assay demonstrate a significant increase in the frequency of heartbeats in the presence of Rhopr-SIFa. In *R. prolixus* cardiovascular activity has been shown to increase under a number of physiological stressors, such that increased heartbeat frequency accelerates hemolymph circulation after a large blood meal, promoting distribution of neurohormones and nutrients (Hamoudi et al., 2016), and enhancing the insect’s ability to perceive environmental stimuli (Chiang et al., 1992). These findings support at the least a peripheral role for Rhopr-SIFa, but also is suggestive of a possible neurohormonal role whereby Rhopr-SIFa may be released from the CC and dorsal vessel, and act peripherally on the posterior dorsal vessel, which controls heartbeat frequency. Since the SIFa receptor has been shown to be expressed in peripheral tissues in *B. terrestris* (Lismont et al., 2018) it will be critical to determine if the same is true for the receptor in *R. prolixus*, since a Rhopr-SIFa receptor has been predicted in the *R. prolixus* genomic sequence (Ons et al., 2016).

We show that Rhopr-SIFa, similar to gonadotropin inhibitory hormone(GnIH) in vertebrates (Clarke et al., 2012; McConn et al., 2014), promotes food intake in *R. prolixus*. The SIFaR is a homolog of the vertebrate GnIH receptor (GnIHR), although their respective ligands, SIFamide and GnIH, are not sequence-related (Jørgensen et al., 2006; Jékely, 2013; Ubuka and Tsutsui, 2014). GnIHR regulates food intake and reproductive processes and has been shown to promote feeding behavior over other kinds of behavior in periods of metabolic needs (Clarke et al., 2012). However, it remains unclear whether the functions of the SIFamide- and GnIH-signaling pathways are conserved across phyla. Our results do suggest the presence of an evolutionarily conserved signaling system across vertebrates and arthropods in relation to food intake. The present work also shows, for the first time, that Rhopr-SIFa is released outside of the CNS and alters hemolymph circulation in *R. prolixus*. The mechanisms by which Rhopr-SIFa exerts these effects are yet to be investigated. Future work utilizing molecular techniques to explore Rhopr-SIFa receptor expression and its effects on physiology of potential target tissues will be of particular interest. It is likely that a variety of neuropeptides, including Rhopr-SIFa, establish a network of communication and feedback to process hunger and satiety signals. Understanding the Rhopr-SIFa pathway, its components and mechanisms of action, has implications for vector control by highlighting targets to alter feeding, diuresis, and the spread of this disease vector.

## Data Availability Statement

The raw data supporting the conclusions of this article will be made available by the authors, without undue reservation, to any qualified researcher.

## Author Contributions

MA designed and performed all experiments, data analysis, and written work. MH initiated early aspects of this research and completed exploratory work. AL contributed to experimental design and revisions to manuscript. IO contributed to experimental design and revisions to manuscript.

## Conflict of Interest

The authors declare that the research was conducted in the absence of any commercial or financial relationships that could be construed as a potential conflict of interest.

## Footnotes

1 www.graphpad.com

2 www.ch.embnet.org/software/BOX_form.html
