# Supplementary material for: SIFamide Influences Feeding in the Chagas Disease Vector, Rhodnius prolixus
Source: Front Neurosci. 2020 Feb 21;14:134. doi: 10.3389/fnins.2020.00134 (PMC7047498; doi:10.3389/fnins.2020.00134)
Supplement: Supplementary file 2 [file Image_2.pdf]

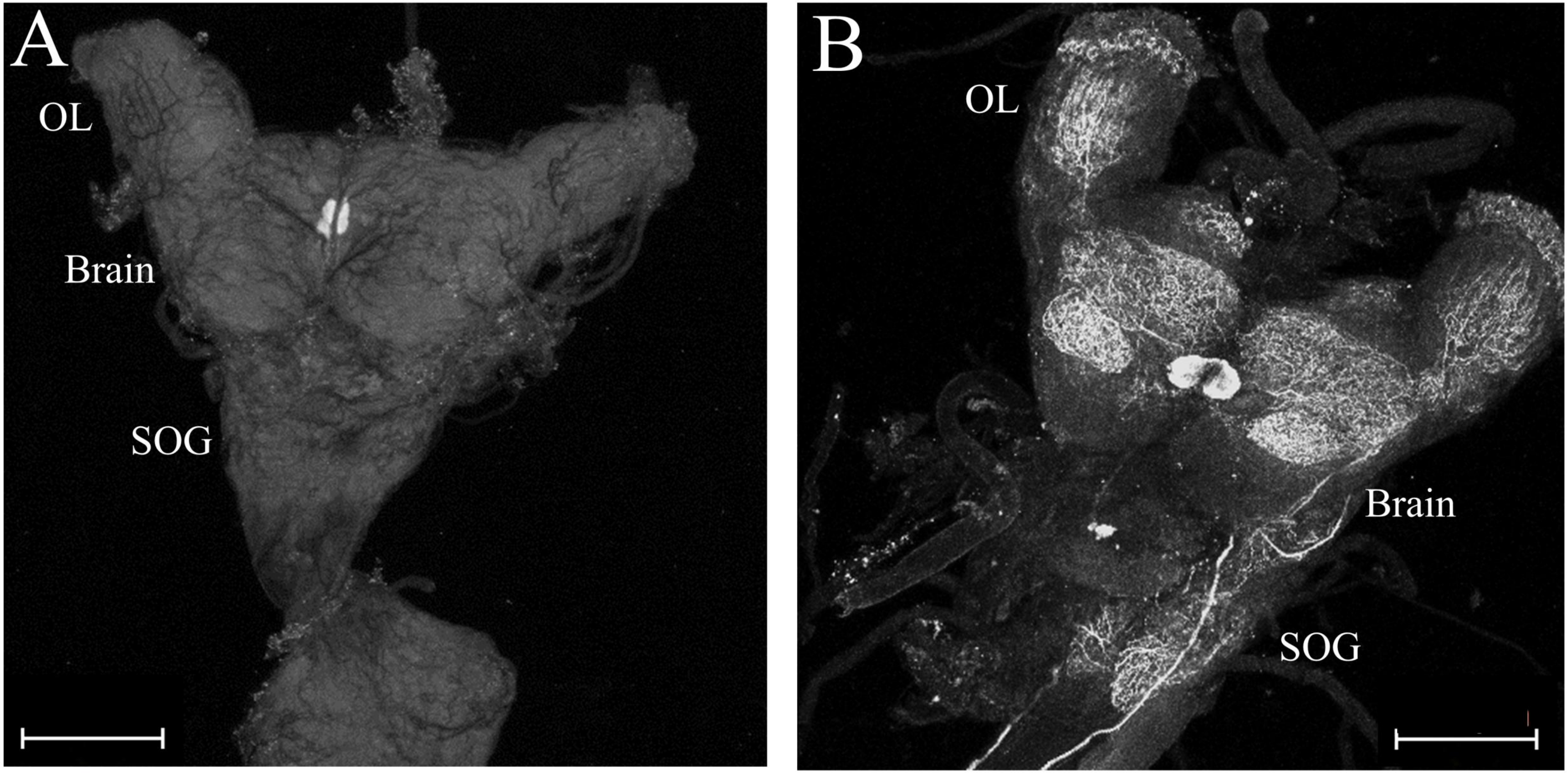

Supplementary Figure 2: Verification of antibody specificity for Rhopr-SIFa. Central nervous systems from 5th instar insects were incubated in antiserum that had been pre-absorbed with 10^{-5} M Rhopr-SIFa or in antiserum without pre-absorption. **(A)** Preabsorbed antiserum resulted in reduced staining intensity in cell bodies and an absence of staining in neuropile processes. (B) Preparations (without pre-absorption with Rhopr-SIFa) display bright SIFa-like immunoreactive staining in cell bodies and neuropile processes. Brain; SOG, suboesophageal ganglion; PRO, prothoracic ganglion; OL, optic lobe. Scale bars: 100 μm.
