# Supplementary material for: SIFamide Influences Feeding in the Chagas Disease Vector, Rhodnius prolixus
Source: Front Neurosci. 2020 Feb 21;14:134. doi: 10.3389/fnins.2020.00134 (PMC7047498; doi:10.3389/fnins.2020.00134)
Supplement: Supplementary file 3 [file Image_3.pdf]

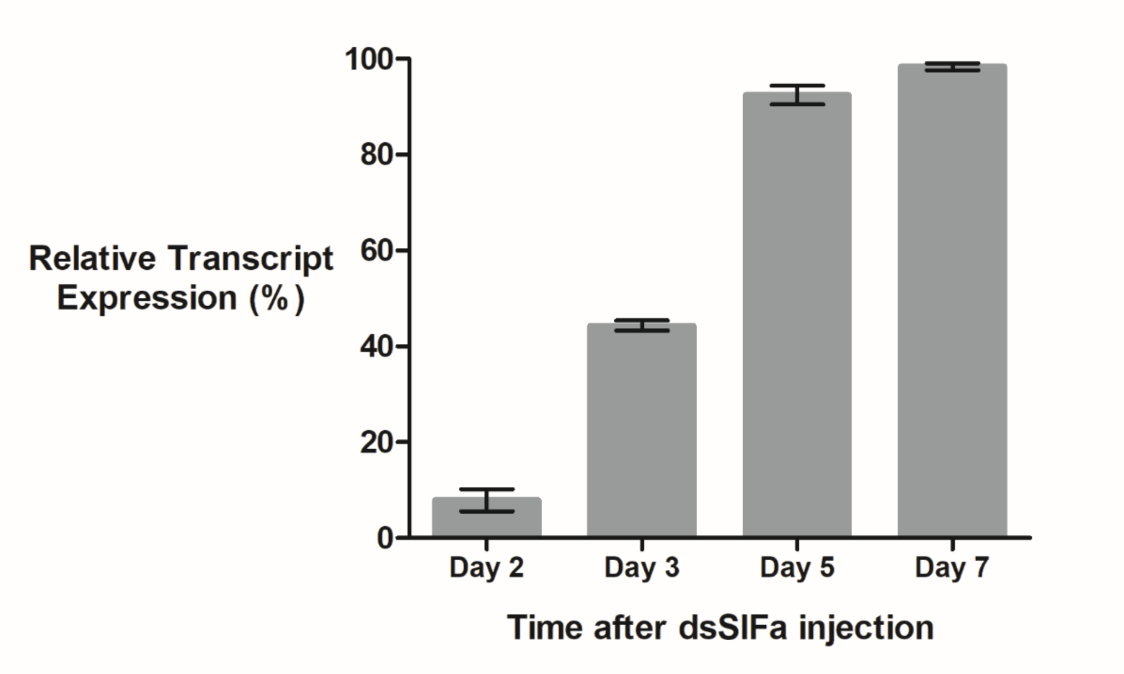

Supplementary Figure S3: Verification of dsRNA efficiency by real time qPCR. Rhopr-SIFa transcript expression levels were reduced in 5th instar central nervous system (CNS) following injection of dsSIFa, relative to control dsARG injection. Day 2 after injection with dsSIFa Rhopr-SIFa transcript levels were reduced by 92% compared to dsARG injected controls. Error bars represent standard error of the mean.
