# Supplementary material for: SIFamide Influences Feeding in the Chagas Disease Vector, Rhodnius prolixus
Source: Front Neurosci. 2020 Feb 21;14:134. doi: 10.3389/fnins.2020.00134 (PMC7047498; doi:10.3389/fnins.2020.00134)
Supplement: Supplementary file 4 [file Image_4.pdf]

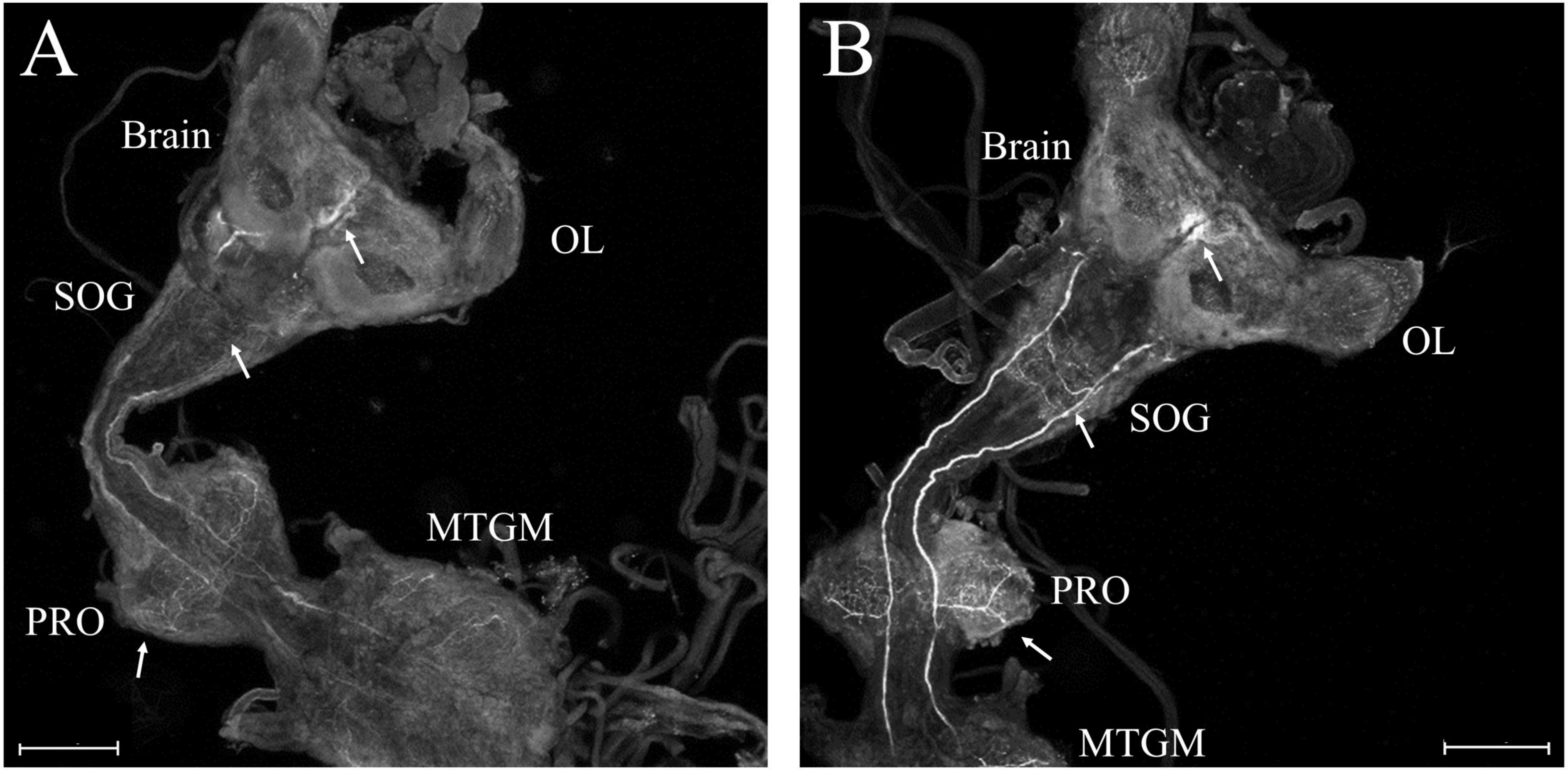

Supplementary Figure 4: Verification of dsRNA knockdown by immunohistochemistry. Central nervous systems from insects injected with dsSIFa or dsARG were examined Day 2 post-injection. **(A)** dsSIFa-treated insects had reduced staining intensity in cell bodies and neuropile processes (arrows). (B) dsARG-injected insects display bright SIFa-like immunoreactive staining in cell bodies and neuropile processes (arrows). Brain; SOG, suboesophageal ganglionic mass; PRO, prothoracic ganglion; MTGM, mesothoracic ganglionic mass; OL, optic lobe. Scale bars: 100 µm
