# Supplementary material for: SIFamide Influences Feeding in the Chagas Disease Vector, Rhodnius prolixus
Source: Front Neurosci. 2020 Feb 21;14:134. doi: 10.3389/fnins.2020.00134 (PMC7047498; doi:10.3389/fnins.2020.00134)
Supplement: Supplementary file 5 [file Table_1.pdf]

Supplemental Table 1. Gene-specific primers used for Rhopr-SIFamide cDNA isolation and modified 5' and 3' RACE PCR and plasmid vector primers.

**Oligo Name**	**Oligo Sequence (5'-3')**
SIFa-Fw	CATGTCTCGCACTCTGTTCG
SIFa Rv	GTTGTCTTGAACGGGAAACC
SIFa Fw3	GAAGCCTGTTCTGCCTGG
LIB Rev25	GCCAAACCAATGGTCTAGAAAG
SIFa Rv3	AGGGTGAAGCAGCAGACG
LIB Fw1	GTGGATAACCGTATTACCGCC
