# Supplementary material for: SIFamide Influences Feeding in the Chagas Disease Vector, Rhodnius prolixus
Source: Front Neurosci. 2020 Feb 21;14:134. doi: 10.3389/fnins.2020.00134 (PMC7047498; doi:10.3389/fnins.2020.00134)
Supplement: Supplementary file 6 [file Table_2.pdf]

Supplemental Table 2: Primers used to construct dsRNA for A: Rhopr-SIFa expression knockdown. B: dsARG for control injections.

**A: Primers used to generate dsSIFa (5' – 3')**	
SIFa-Fw	CATGTCTCGCACTCTGTTCG
SIFa Rv	GTTGTCTTGAACGGGAAACC
SIFa Fw T7	**TAATACGACTCACTATAGGGAGA**CATGTCTCGCACTCTGTTCG
SIFa Rv T7	**TAATACGACTCACTATAGGGAGA**GTTGTCTTGAACGGGAAACC
**B: Primers used to generate dsARG (5' – 3')**	
dsRNA-ARG-FOR1	ATGAGTATTCAACATTTCCGTGTC
dsRNA-ARG-REV2	AATAGTTTGCGCAACGTTG
dsRNA-ARG-FOR1 T7	**TAATACGACTCACTATAGGGAGA**ATGAGTATTCAACATTTCCGTGTC
dsRNA-ARG-REV2 T7	**TAATACGACTCACTATAGGGAGA**AATAGTTTGCGCAACGTTG

*** TAATACGACTCACTATAGGGAGA** = T7 RNA polymerase promoter region
