# Supplementary material for: SIFamide Influences Feeding in the Chagas Disease Vector, Rhodnius prolixus
Source: Front Neurosci. 2020 Feb 21;14:134. doi: 10.3389/fnins.2020.00134 (PMC7047498; doi:10.3389/fnins.2020.00134)
Supplement: Supplementary file 1 [file Image_1.pdf]

5’---tct*tga*acgggaaaccaggcagaacaggcttcggcagcataactct

tc*taa*tacgaaccacta*tag*ggcaagcagtggtatcaacgcagagtacatggggatcagtcgatccaacgacttccaaccaaactctcttttagaaccaaccagaacaa

Intron 1: 2,501 bp

aacccaccgaccatc atg tct cgc act ctg ttc gtc tgc tgc ttc acc ctg gtc gtc gcc ctg atc
M S R T L F V C C F T L V V A L I 17

ttc ttg gac gct gcc atg gct acc tac aag aaa cca ccc ttc aat ggc agc atc ttc ggc
F L D A A M A T Y K K P P F N G S I F G 37

Intron 2: 74 bp

aag aga gcc ggc cca tct tcc gac tat gaa acc gca gga aaa gct ctg tcc acc atg tgc
K R A G P S S D Y E T A G K A L S T M C 57

gaa att gct gcc gaa gcc tgt tct gcc tgg ttt ccc gtt caa gac aac aat taa ggcaacttt
E I A A E A C S A W F P V Q D N N * 74

tccccttcattttccagactatgaaaccgcaggaaaagctctgtccaccatgtgcgaaattgctgccgaagcctgttctgcctggtttcccgttcaagacaacaatcga

attcccgcggccgccatggcggccggag*taa*ttttcatttttttgaactattacaatagtactaac*taa*ttggaaaatgtattaataaatttatttaaaatttcaaaa

aaaaaaaaaaaaaaaaaaaaaaaaacaggg ---‘3

Supplementary Figure 1. Complete nucleotide sequence of cDNA encoding SIFamide in *R. prolixus.* Nucleotide and amino acid sequences of the coding region start at the nucleotide sequence ATG and asterisk indicates stop codon terminating the ORF (indicated by blue frame). The grey shaded region indicates the mature Rhopr-SIFa amino acid sequence. The length of the sequence previously reported (Ons et al., 2011; GenBank Accession No. ACT35307.1) is indicated by solid arrows. A predicted dibasic proteolytic cleavage site KR is underlined. Open arrows indicate position and length of introns. Stop codons in 3’ and 5’ UTR are italicized.
